# Femoral rotational osteotomy for posterior hip impingement in young adults with increased femoral version

**DOI:** 10.1007/s00264-025-06646-6

**Published:** 2025-08-29

**Authors:** Khaled M. Emara, Ramy Ahmed Diab, Ahmed K. Emara, Mohamed Eissa, Ahmed Ezzat, Mohamed Amr Hemida, Hassan Abdel Hamid Abdel Fattah

**Affiliations:** 1 Ain Shams University, Cairo, Egypt; 2 Cleveland Clinic, Cleveland, USA

**Keywords:** Femoral version, Rotational osteotomy, Hip impingement

## Abstract

**Purpose:**

Posterior femoro-acetabular impingement in patients with increased femoral version can result in significant hip pain, chondro-labral injury, and limited range of motion. Femoral rotational osteotomy may address these issues by correcting excessive femoral anteversion.

**Methods:**

This retro-spective case series included 25 adolescents (mean age 14.8 years) with symptomatic increased femoral version (> 35°) treated between 2015 and 2022. Inclusion required hip pain, limited range of motion, and increased femoral version confirmed on computed tomography. Patients underwent femoral external rotational osteotomy targeting a post-operative femoral version of ~ 15°. Outcomes assessed included femoral version, hip range of motion, and Harris Hip Score pre-operatively, at six months, and at two years post-operatively.

**Results:**

Mean femoral version improved significantly from 39° ± 3° pre-operatively to 19° ± 7° post-operatively (*P* < 0.001). Internal rotation decreased from 54° ± 9° to 32° ± 8°, while external rotation increased from 38° ± 4° to 44° ± 5° (*P* < 0.001). Mean Harris Hip Score improved from 62.5 ± 10.3 to 86.1 ± 6.4 at 6 months, with sustained results at two year follow-up. Radiographic union was achieved in all patients, and no major complications were observed.

**Conclusion:**

Femoral rotational osteotomy is a safe and effective treatment for posterior hip impingement in young patients with excessive femoral version.

## Introduction

Intra-articular posterior femoro-acetabular impingement (PFAI) is recognized as a cause of hip pain, chondro-labral damage, and the development of osteoarthritis (OA) in patients with excessive femoral ante-version (AV) [1–3], additionally, the antero-superior portion of the joint experiences overload due to partial uncovering of the femoral head [4, 5].

Extra-articular posterior impingement further contributes to the pathology and is exacerbated by external rotation during both hip flexion and extension. This impingement typically occurs between the ischium and the lesser trochanter during extension, and between the ischium and the greater trochanter or inter-trochanteric region during flexion [6].

Abnormal contact has also been described between the extra-capsular femoral neck and adjacent bony structures, such as the ilium, ischium, anterior inferior iliac spine, or acetabular rim [7–9].

Patients often report pain during hip flexion, abduction, and external rotation. Some may also experience sciatica-like symptoms due to compression of the sciatic nerve located just posterior to the quadratus femoris muscle [10, 11].

Patients with femoral version abnormalities reported markedly worse quality of life as measured on the Limb Deformity-Modified Scoliosis Research Society and Patient-Reported Outcomes Measurement Information System questionnaire scores compared with healthy control subjects [12].

Correction of femoral torsional deformities is recently targeted at younger age and by novel techniques as rotational guided growth, and leads to functional gait improvement [13] and hip arthroscopy alone cannot solve all impingement problems [14], in addition external rotation osteotomy leads to significant increases the ischio-femoral space [15].

Rigling et al. investigated the clinical and radiological results of subtrochanteric rotational osteotomy as a treatment option for symptomatic femoral maltorsion and its potential adverse effects, particularly on patellofemoral stability and geometry, they noted a significant improvement of Subjective Hip value (SHV) and Harris Hip Score (HHS) without leading to objective patellofemoral instability nor changes in the patellofemoral geometry compared to contralateral side [16].

Foot-progression-angle showed significant improvement and patients walked with less In-toeing after femoral osteotomy as reported by Lerch et al. in addition to the significant increase in SHV [17].

Our hypothesis is that femoral rotational osteotomy can improve hip pain, range of motion, and overall function in symptomatic young adults with increased femoral AV.

## Materials and methods

This retro-spective study included 25 consecutive patients who underwent femoral rotational osteotomy between 2015 and 2022. All patients presented with symptomatic increased AV, manifested as recurrent hip pain. Diagnosis was confirmed through clinical examination, including range of motion (ROM), and several standard tests: the anterior impingement test [18] (pain in forced flexion, internal rotation, and adduction), the posterior impingement test [19] (pain in forced extension and external rotation).

Standardized antero-posterior and lateral radiographs were obtained for all patients. CT scan was used to measure femoral version applying the method described by Murphy et al. [20]. Increased AV was defined as greater than 35° [21]. AV was measured pre-operative and six months post-operative.

HHS [22] and ROM parameters were evaluated pre-operatively, at six months post-operatively, and 2 years post-operative. Radiographic union and complications were also recorded.

Inclusion Criteria: 1- Age between 12 and 16 years. 2- Posterior hip pain with clinical signs of posterior impingement. 3- FV > 35° on CT. 4- Failure of conservative treatment for > six months. Exclusion Criteria: 1- Moderate to severe hip OA as per the Tönnis grading system [23]. 2- Previous proximal femoral or pelvic surgery. 3- Cam lesion or hip dysplasia on preoperative X-ray or CT.

All surgeries were performed by a single surgeon at a tertiary institutional hospital. The study adhered to the principles of the 1964 Declaration of Helsinki and its subsequent amendments and was approved by the institutional ethics and research committee.

Patients were positioned supine on a radiolucent table. A direct lateral approach was used to expose the femur, through a subvastus approach. The level of osteotomy—consistently at the diaphyseal level—was determined under image intensification. A dynamic compression plate (narrow or broad) was selected based on patient anatomy. The osteotomy was initiated with multiple drill holes to weaken the cortex and then completed using an osteotome. The aim was to externally rotate the distal femur to achieve a post-operative AV of approximately 15°. The distal fragment was rotated to match the pre-determined correction. After fixation with the plate, the surgical field was thoroughly irrigated, and tissues were closed in layers. Patients were kept non-weight-bearing for four weeks. This was followed by a structured physical therapy regimen focusing on gait training, balance, and abductor muscle strengthening. Partial weight-bearing with crutches was initiated after this period, progressing to full weight-bearing without assistance guided by plain X-ray that was done monthly till sixth months post-operative and at two years post-operative.

Follow-up assessments were conducted at regular intervals: weekly for the first month, biweekly for the second month, monthly for the subsequent four months, and every three months thereafter. All patients were followed for a minimum of two years.

## Results

Mean patient age was 14.8 years (range, 12–16). Twenty-five patients were included (12 boys and 13 girls), with a mean follow-up of 2.3 years (range, 2–4 years).

**Fig. 1:**
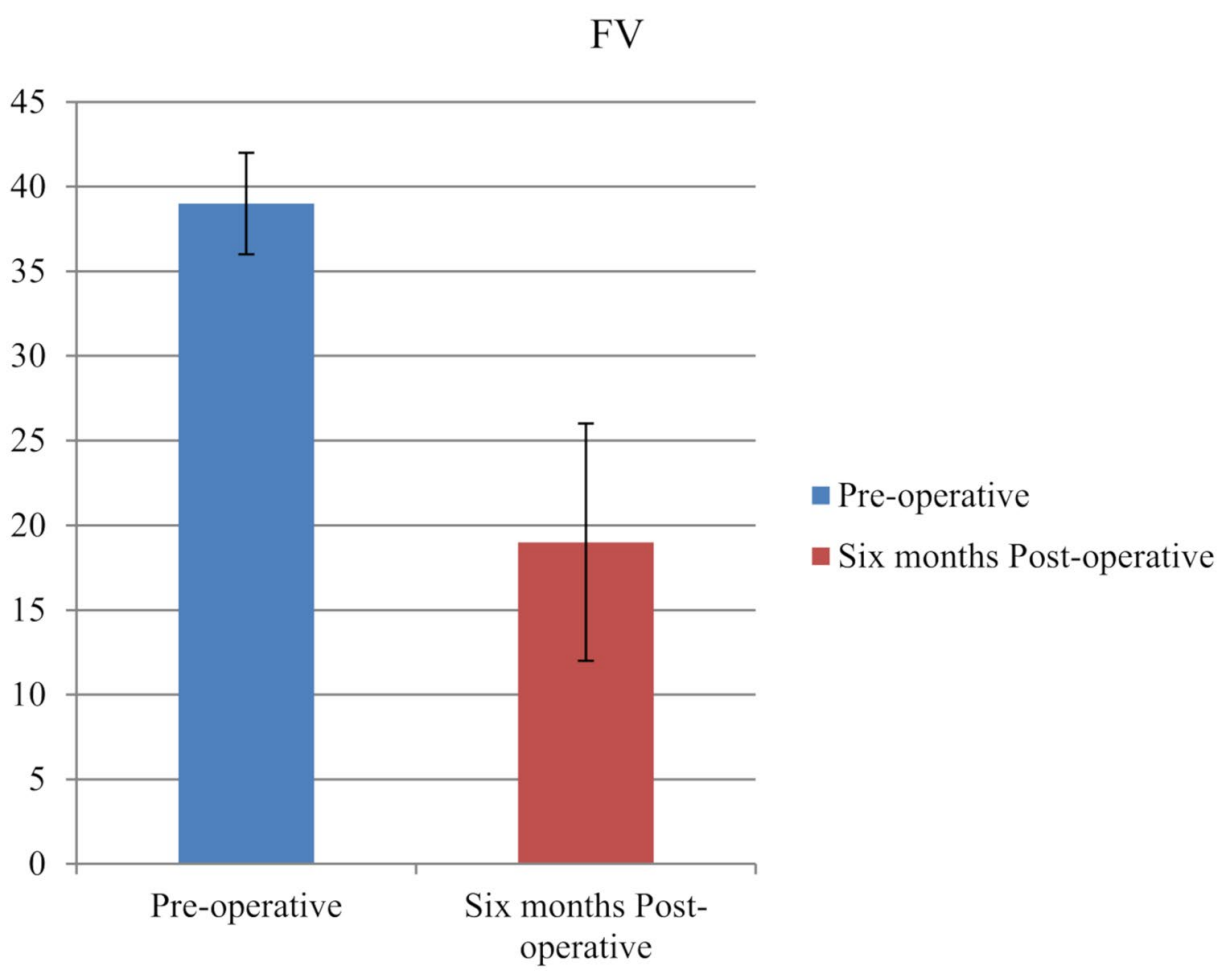
Pre-operative and 6-month post-operative comparison of femoral version (FV). A significant reduction was observed following femoral rotational osteotomy (*P* < 0.001)

Preoperative femoral version averaged 42.6° (SD 5.4), which was corrected to 18.2° (SD 4.1). Average follow-up duration was 30 months (range, 24–36). AV decreased significantly from 39° (SD 3) pre-operatively, to 19° (SD 7) post-operatively (*P* < 0.001; Fig. 1).

**Fig. 2:**
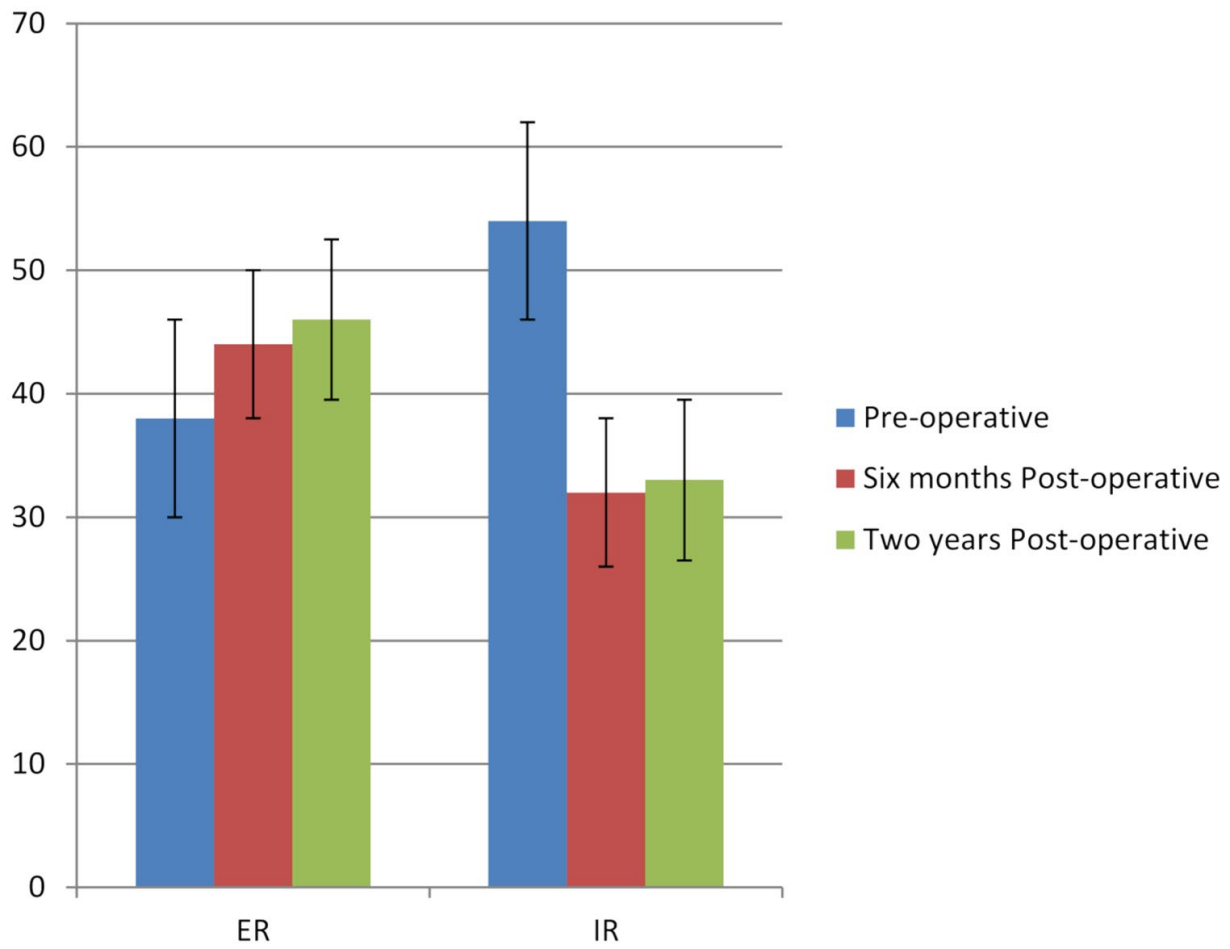
Changes in external rotation (ER) and internal rotation (IR) from preoperative to 6-month and 2-year postoperative follow-up. ER significantly increased, while IR significantly decreased postoperatively (*P* < 0.001)

Internal rotation decreased from 54° (SD 9), to 32° (SD 8), while external rotation improved from 38° (SD 4), to 44° (SD 5) (*P* < 0.001 for both; Fig. 2). Other ROM parameters showed minimal, non-significant changes (Table 1).

**Table 1:** Comparison of preoperative, 6-Month, and 2-Year postoperative outcomes. Values are presented as mean ± SD. Statistical comparisons were performed using paired sample t-tests

Parameter	Preop.	6-Month Postop.	2-Year Postop.	*P* Value (Pre vs 6 M.)	*P* Value (Pre vs 2 Yr)
**Femoral Version (°)**	39 ± 3	19 ± 7	—	< 0.001**	—
**Range of Motion (°)**					
Flexion	105 ± 10	110 ± 8	110 ± 10	0.057	0.084
Extension	9 ± 6	11 ± 7	12 ± 7	0.284	0.110
Abduction	35 ± 8	37 ± 9	39 ± 7	0.830	0.066
Adduction	25 ± 3	25 ± 3	25 ± 3	1.000	1.000
External Rotation	38 ± 4	44 ± 5	46 ± 2	< 0.001**	< 0.001**
Internal Rotation	54 ± 9	32 ± 8	33 ± 9	< 0.001**	< 0.001**
**Harris Hip Score**	62.5 ± 10.3	86.1 ± 6.4	88.2 ± 6.1	< 0.001**	< 0.001**

Using Paired sample t-test

**P < 0.001, highly significant

**Fig. 3:**
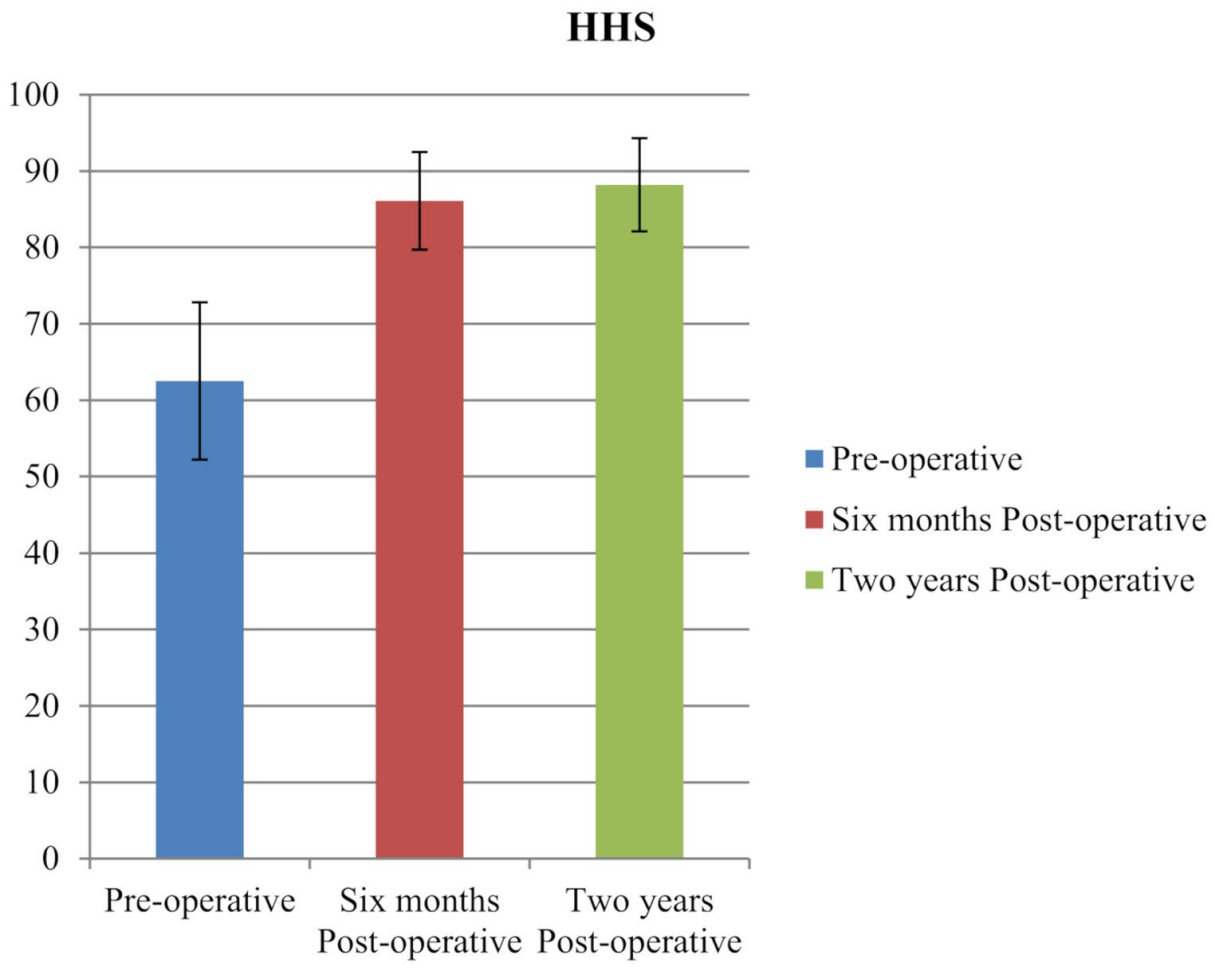
Improvement in Harris Hip Score (HHS) from preoperative to 6-month and 2-year postoperative assessments, demonstrating sustained functional gains following surgery (*P* < 0.001)

The Harris Hip Score (HHS) improved from a preoperative mean of 62.5 (SD10.3) to 86.1 (SD 6.4) at six months postoperatively (*P* < 0.001; Fig. 3). These improvements were maintained at two year follow-up with minimal variation. Significant differences were observed between pre-operative, six month, and two year post-operative measurements for AV, external rotation, internal rotation, and HHS (*P* < 0.001 for all comparisons).

Post-operative complications were minimal. Two patients developed superficial wound infections, successfully managed with local wound care and parenteral antibiotics. One patient required hardware removal due to persistent discomfort. No cases of nonunion, hardware failure, or additional reoperations were reported. Radiographic union was confirmed at a mean of 3.2 months postoperatively, suggesting the osteotomy technique is reliable for achieving bony healing.

### Case presentation

A 13-year-old girl presented with intoeing gait and bilateral recurrent hip pain. Clinical examination revealed increased AV and a positive impingement test. ROM was within normal limits, except for increased internal rotation and a painful limitation of external rotation to 34°. HHS was 72 preoperatively, and AV measured 37°. A right-sided femoral osteotomy was performed and fixed with a narrow dynamic compression plate. Post-operatively, AV was corrected to 23°. At six months, the HHS had improved to 86, with external rotation measuring 40°. At the two-year follow-up, the HHS was 88, and external rotation measured 43°. The patient was scheduled for a similar procedure on the left side.

## Discussion

The optimal treatment of hips with increased AV remains controversial. While some authors advocate for open correction using proximal femoral osteotomies [5, 24] to reduce AV, others have explored arthroscopic techniques, including femoral cam resection and lesser trochanter resection [25, 26]. However, the efficacy of these arthroscopic interventions in improving pain and ROM in patients with increased AV has not been definitively established. Importantly, arthroscopic cam resection may fail to address underlying posterior hip impingement and abnormal femoral version, potentially limiting clinical improvement [27]. Similarly, the management of extra-articular hip impingement is debated, with both open and arthroscopic approaches currently in use [28].

A recent systematic review identified persistent deformity and inadequate correction as significant risk factors for suboptimal patient-reported outcomes following hip arthroscopy [29]. These same factors are among the most frequent indications for revision procedures in the setting of PFAI [29, 30].

In our study, we focused on symptomatic adolescents with increased AV who lacked intra-articular cam lesions. All patients underwent an isolated extra-articular derotation osteotomy, enabling us to specifically assess the benefits of femoral version correction on hip pain.

Pailhé et al. reported outcomes in six adolescents (mean age, 13.6 years) who underwent nine derotation osteotomies to address excessive AV. The procedure, performed via a distal supracondylar osteotomy stabilized with an antegrade intramedullary nail, achieved an average correction of 19°. All patients reported satisfaction and demonstrated improvements in foot progression angles and internal rotation on examination [31].

Similarly, Robert et al. described closed sub-trochanteric derotation osteotomy as a safe and effective technique for correcting both femoral retro-version and excessive ante-version. Fixation was performed with a variety of intra-medullary nails. Excellent or good outcomes were reported in 93% of cases, with statistically significant improvements in the modified Harris Hip Score. Although generally better tolerated than a blade plate after inter-trochanteric osteotomy more than two-thirds of patients required implant removal due to thigh discomfort or screw irritation [32], the diaphyseal location of the implant in our study minimized hardware prominence.

Intra-medullary nailing carries risks, including potential damage to the hip abductors during canal reaming. However, its advantages include immediate weight bearing, due to the inherent stability provided by locked intramedullary fixation. The use of dynamic distal interlocking screws may also promote compression at the osteotomy site during ambulation [32]. In contrast, plating typically necessitates restricted weight bearing for at least six weeks postoperatively.

Femoral diaphyseal derotation osteotomy has proven to be a safe and reliable technique. In our series, there were no cases of non-union or hardware failure. Despite the smaller surface area of a transverse osteotomy and the relatively lower healing potential of diaphyseal cortical bone compared to cancellous bone, all osteotomies united successfully.

It is important to consider tibial maltorsion as an aetiology of hip pain. Tibial derotation osteotomy results in significant clinical and functional recovery within 12 months in symptomatic hip impingement patients even in the presence of co-existing pathomorphology [33].

This study has several limitations. First, the absence of a control group limits the ability to compare outcomes with other treatment modalities. This should be addressed in future research. Second, approximately 13% of patients with increased AV may also have compensatory external tibial torsion. In such cases, externally rotating the distal femoral segment could exacerbate an already increased external foot progression angle [32]. We used a threshold of > 35° to define increased AV, but definitions vary across the literature, and alternative thresholds could potentially yield different findings.

Another point is that the ischio-femoral space was not measured in this study, it has been proven that supra-condylar external rotation osteotomy of the femur leads to a significant increase in the ischio-femoral space [34].

Additionally, patients with dysplastic hip morphology and cam lesions were excluded. Future studies should investigate combined surgical approaches, such as femoral derotation osteotomy with periacetabular osteotomy or hip arthroscopy, to address more complex pathologies involving both intra- and extra-articular components. Recently, hip shelf acetabuloplasty has emerged as a simple and effective treatment option for borderline hip dysplasia, with favourable outcomes [35]. This procedure may be readily combined with femoral derotation osteotomy in cases of combined pathology and warrants further investigation in future studies.

Another relevant clinical scenario involves the coexistence of slipped capital femoral epiphysis and excessive femoral version in adolescents. In such cases, incorporating a derotation component into the modified trochanteric triplane osteotomy may be beneficial. This technique has recently been recognised as a safe and effective treatment option for chronic moderate to severe slips [36] and may provide a comprehensive solution in complex deformities.

## Conclusion

Femoral rotational osteotomy is a safe and effective treatment for posterior hip impingement in young patients with excessive femoral version.

## Data Availability

No datasets were generated or analysed during the current study.
